# The RAGE Inhibitor TTP488 (Azeliragon) Demonstrates Anti-Tumor Activity and Enhances the Efficacy of Radiation Therapy in Pancreatic Cancer Cell Lines

**DOI:** 10.3390/cancers17010017

**Published:** 2024-12-24

**Authors:** Kumari Alka, Jacob F. Oyeniyi, Ghulam Mohammad, Yi Zhao, Stephen Marcus, Prakash Chinnaiyan

**Affiliations:** 1 Department of Radiation Oncology, Corewell Health William Beaumont University Hospital, Royal Oak, MI 48076, USA; kumari.alka@corewellhealth.org (K.A.); jacob.oyeniyi@corewellhealth.org (J.F.O.); ghulam.mohammad@corewellhealth.org (G.M.); yi.zhao@corewellhealth.org (Y.Z.); 2 Cantex Pharmaceuticals, Weston, FL 33326, USA; 3 Radiation Oncology, Oakland University William Beaumont School of Medicine, Rochester, MI 48309, USA

**Keywords:** pancreatic cancer, RAGE, Azeliragon, NF-κB, immunosuppressive microenvironment, radiation therapy

## Abstract

Pancreatic cancer is the third leading cause of cancer-related mortality in the United States, with rising incidence and mortality. The receptor for advanced glycation end products (RAGE) and its ligands significantly contribute to pancreatic cancer progression by enhancing cell proliferation, fostering treatment resistance, and promoting a pro-tumor microenvironment via activation of the nuclear factor-kappa B (NF-κB) signaling pathways. This study validated pathway activation in human pancreatic cancer and evaluated the therapeutic efficacy of TTP488 (Azeliragon), a small-molecule RAGE inhibitor, alone and in combination with radiation therapy (RT) in preclinical models of pancreatic cancer. Human (Panc1) and murine (Pan02) pancreatic cancer cell lines exhibited elevated levels of RAGE and its ligands compared to normal pancreatic tissue. In vitro, Azeliragon inhibited RAGE-mediated NF-κB activation and ligand-mediated cell proliferation in pancreatic cancer cell lines. Target engagement of Azeliragon was confirmed in vivo, as determined by decreased NF-κB activation. Azeliragon demonstrated significant growth delay in mouse models of pancreatic cancer and additive effects when combined with RT. Additionally, Azeliragon modulated the immune suppressive tumor microenvironment in pancreatic cancer by reducing immunosuppressive cells, including M2 macrophages, regulatory T cells, and myeloid-derived suppressor cells, while enhancing CD8+ T cell infiltration. These findings suggest that Azeliragon, by inhibiting RAGE-mediated signaling and modulating immune response, may serve as an effective anti-cancer agent in pancreatic cancer.

## Simple Summary

Pancreatic cancer is a leading cause of cancer-related deaths in the U.S. The receptor for advanced glycation end products (RAGE) plays a key role in this disease by promoting tumor growth, treatment resistance, and suppressing immune response. This study investigated TTP488 (Azeliragon), a RAGE inhibitor, both alone and combined with radiation therapy (RT) in models of pancreatic cancer. In laboratory and animal tests, Azeliragon blocked RAGE-related pathways, reduced tumor growth, enhanced RT response, and modulated the immune-suppressive tumor microenvironment in pancreatic cancer. These findings suggest that Azeliragon, used alone or in combination with RT, could be a valuable treatment approach for pancreatic cancer and warrants further investigation.

## 1. Introduction

Pancreatic cancer is the third leading cause of cancer deaths in the United States, contributing to 3% of cancer incidence and 8% of cancer mortality, with an expected increase in incidence and mortality over the next decade. It is projected to become the second leading cause of cancer-related death by 2030 [1,2]. The five-year survival rate for patients with pancreatic cancer has improved marginally over the past two decades and is currently estimated to be 10%, 3%, 15%, and 36% for all stages, metastatic, regional, and localized disease, respectively [1]. The tumor microenvironment has been attributed to the observed therapeutic resistance in this malignancy, wherein hypoxia, immunosuppression, and the accumulation of anti-apoptotic proteins are predominant features [3,4,5]. Novel treatment strategies, including those aimed at modulating the tumor microenvironment, hold promise for achieving clinical gains in this aggressive malignancy.

The receptor for advanced glycation end products (RAGE) is a transmembrane receptor belonging to the immunoglobulin superfamily that has been implicated in various pathological processes, including cancer, diabetes, and neurodegenerative conditions such as Alzheimer’s disease [6,7,8,9]. It binds to a diverse array of ligands, such as advanced glycation end products (AGE), Mac-1, S100 proteins, HMGB1, glycosaminoglycans, and amyloid-β-proteins. The activation of RAGE by these ligands triggers diverse signaling pathways, which vary based on factors including the ligand type, concentration, and cellular context [6,7]. This versatility in ligand recognition has led to RAGE being classified as a pattern recognition receptor. Additionally, because many RAGE ligands are linked to tissue damage and stress, RAGE is also described as a receptor for damage-associated molecular patterns (DAMPs), and upregulated RAGE protein in human tumors portends a higher histological grade and poorer clinical outcomes in multiple cancers [10,11,12,13,14,15].

RAGE has been implicated in the development, advancement, and treatment resistance of pancreatic cancer. DiNorcia et al. demonstrated that deletion of the RAGE gene inhibits pancreatic tumor development and significantly prolongs survival in a mouse model [16]. RAGE has been shown to promote growth and survival in pancreatic cancer cells via multiple mechanisms, including sustaining autophagy, limiting apoptosis, and providing protection against oxidative stress, both in vivo and in vitro. Additionally, RAGE enhances tumor cell ATP production, proliferation, and migration [17,18,19]. Similarly, up-regulation of RAGE in pancreatic cancer cells has been shown to differentially promote cell proliferation and migration [20].

In addition to the receptor itself, RAGE ligands such as S100P and high mobility group box 1 (HMGB1) have been widely investigated for their roles in pancreatic cancer [21,22,23]. S100P has been shown to enhance cell proliferation and migration in pancreatic cancer Panc-1 cells through a RAGE-dependent mechanism. Elevated levels of S100P correlate with increased proliferation, survival, migration, and invasion in both in vitro and in vivo models, and these effects were inhibited by a RAGE antagonist peptide [21]. Interactions between RAGE and its ligands activate multiple signaling pathways, including MAP kinase and p38 mitogen-activated protein kinase, Ras kinase, Cdc42/Rac, and stress-activated protein kinase, ultimately inducing a sustained NF-κB response [24,25,26,27]. NF-κB is a transcription factor that upregulates anti-apoptotic genes such as A1, A20, XIAP, Bcl-2, and Bcl-XL, leading to the overexpression of anti-apoptotic proteins and the activation of the cellular antioxidant defense system [28,29,30]. This NF-κB-mediated mechanism is particularly relevant in response to ionizing radiation, as it reduces cell death and contributes to radiation resistance. Furthermore, the constitutive activation of NF-κB genes in malignant cells has been linked to increased resistance to radiation therapy [31].

**Figure 1:**
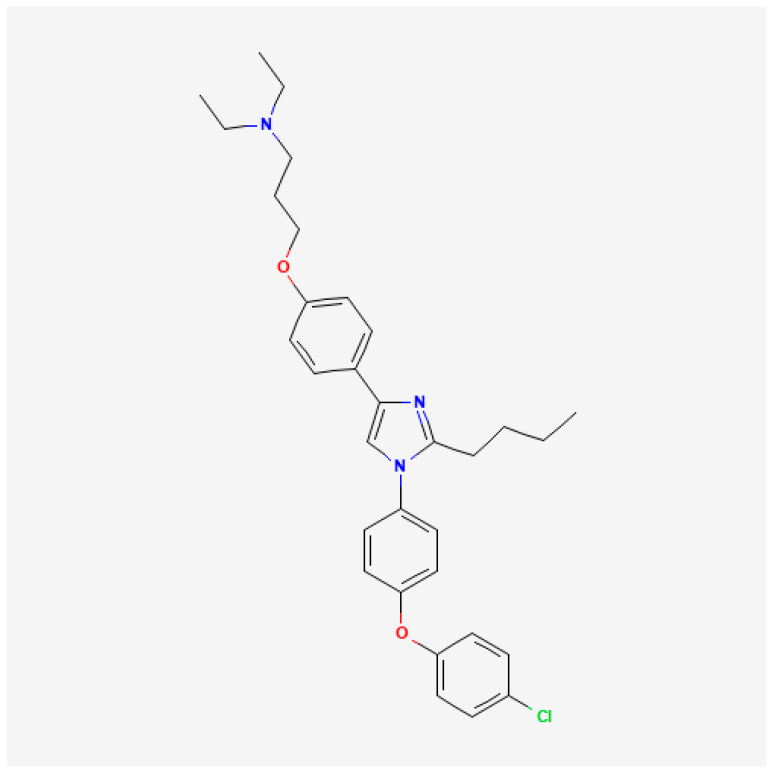
Chemical Structure of Azeliragon (retrieved from PubChem) [34].

RAGE inhibitors, primarily through direct inhibition of the RAGE ligand S100P, have shown antitumor activity in preclinical models of pancreatic cancer [32,33]. TTP488 (Azeliragon; Figure 1), an orally bioavailable small-molecule RAGE inhibitor that binds directly to RAGE and blocks its interaction with all ligands, has been thoroughly investigated in human clinical trials and demonstrated excellent safety in patients with Alzheimer’s disease [34,35]. In preclinical animal models of breast cancer, Azeliragon demonstrated efficacy via RAGE inhibition [36]. This compound is currently undergoing phase I/II clinical studies in patients with metastatic pancreatic cancer refractory to prior treatments, as well as phase Ib/II studies in combination with standard chemoradiation in patients with newly diagnosed glioblastoma [37,38]. Based on the multiple ways the RAGE pathway has been implicated in the progression of pancreatic cancer and its capacity to modulate radiation response, we evaluated both the anti-tumor activity of the RAGE inhibitor Azeliragon and its potential to enhance radiation response in pancreatic cancer preclinical models.

## 2. Materials and Methods

### 2.1. Cell Lines

The murine pancreatic adenocarcinoma cell line Panc02 was obtained from the National Cancer Institute (NIH, MD, USA) and was cultured in RPMI-1640 medium supplemented with 10% FBS, 100 units/mL penicillin, and 100 μg/mL streptomycin. (Gibco-Thermofisher, Grand Island, NY, USA). The human Panc1 adenocarcinoma cell line was obtained from the American Type Culture Collection (ATCC, Manassas, VA, USA) and was cultured in DMEM medium supplemented with 10% FBS, 100 units/mL penicillin, and 100 μg/mL streptomycin (Gibco, Thermo Fisher Scientific, Inc., Waltham, MA, USA). Both cell lines were cultured and maintained in a humidified atmosphere containing 5% CO2/95% air at 37 °C. Human pancreas extract (Santa Cruz Biotechnology, TX, USA) and murine pancreas lysate (collected from the pancreas of female C57BL/6NJ mouse) were used as normal pancreas samples.

### 2.2. Cell Proliferation

Cells (10^5^) were plated for 24 h before Azeliragon was added in serial dilution (0.05–3 μM) to the culture medium. Viable cells were counted at the indicated timepoint using trypan blue (Corning/VWR, Chicago, IL, USA). Azeliragon (Cantex Pharmaceuticals, FL, USA) was dissolved in cell culture-grade dimethyl sulfoxide (DMSO, Sigma-Aldrich, St. Louis, MO, USA) and diluted with PBS for in vitro/in vivo studies.

### 2.3. Tumor Model

Female C57BL/6NJ and NU/J mice (5-week-old) were purchased from The Jackson Laboratory (ME, USA). A total of 2 × 10^6^ Pan02 or Panc1 cells suspended in PBS were subcutaneously injected into the left flank of each mouse. Animals were monitored daily, and tumors were measured with a caliper twice a week. Mice were randomized to four groups (*n* = 5 per group): (1) control; (2) radiation alone; (3) Azeliragon alone; (4) and radiation + Azeliragon. On day 14 post-tumor implantation, the mice were treated with AZ (1 mg/kg) intraperitoneally or vehicular control, and one day later, RT treatment was initiated. AZ treatment continued daily till the mice survived. Before radiation treatment, each mouse was anesthetized and shielded using a lead box to ensure that only the flank was irradiated. Faxitron model CP160 (Faxitron XRay Corp., Wheeling, IL, USA) was used to irradiate mice at a dose of 2 Gy daily for 5 days. Mice were euthanized upon reaching endpoint criteria (1000 mm^3^ for C57BL/6NJ mice and 1500 mm^3^ for NU/J mice). For correlative studies, flank tumors (2 × 10^6^ Pan02 or Panc1 cells) were implanted in different sets of mice (Female C57BL/6NJ and NU/J mice), which were randomly selected when the tumor volume reached approximately 150 mm^3^ and divided into the indicated groups. Azeliragon (1 mg/kg, i.p.) or vehicle control was started on day 14 post-tumor implantation. Mice were euthanized at day 24 post-tumor implantation, and tumors were harvested for further studies. All in vivo experiments were performed according to institutional guidelines and approved by the Institutional Animal Care and Use Committee of Corewell Health.

### 2.4. Clonogenic Assay

Cells (10^5^) were seeded into six-well tissue culture plates. After 24 h, cells were treated with Azeliragon (1µM) for 24 h and exposed to radiation at various doses (2Gy, 4Gy, 6Gy) using the CIX2, XSTRAHL Cabinet Irradiator (Camberley, UK). Plates were incubated for 10 to 14 days for colony formation, and the medium was left unchanged during the duration of the experiment. Colonies were stained with crystal violet, and the number of colonies was calculated.

### 2.5. Western Blot

Western blot was performed using established methods. Briefly, proteins in the whole-cell lysate were resolved on 4–12% Criterion XT Bis-Tris gels (Bio-Rad, CA, USA) and transferred to a nitrocellulose membrane. After blocking, the membrane was incubated overnight at 4 °C with various primary antibodies. This was followed by incubation with a secondary antibody (goat anti-rabbit or goat anti-mouse antibody, Cell Signaling Technology, Beverly, MA, USA). The blot was visualized with a chemiluminescence substrate (Thermo Scientific, Massachusetts, NY, USA), and images were captured using an Odyssey infrared imaging system by Li-Cor (Lincoln, NE, USA) and analyzed with ImageJ software. Antibodies against RAGE (216329), HMGB1 (79823), S100P (133554), S100A2 (109494), pNF-kB (76302), and NF-kB (16502) were purchased from Abcam (Cambridge, MA, USA). Antibodies against vinculin were purchased from Cell Signaling Technology (Danvers, MA, USA). For Western blot, the primary antibodies were used at a dilution of 1:1000. Western blot bands were quantified using densitometric analysis with ImageJ software. Data were normalized to the loading control (vinculin) and presented as mean ± SD from three independent experiments.

### 2.6. Flow Cytometric Analysis

Tumors harvested from mice were dissociated into single-cell suspensions using a combination of DNase I and Collagenase IV (Sigma Aldrich, St Louis, MO, USA). The resulting cell suspensions were washed in FACS buffer, which consisted of PBS supplemented with 0.2% BSA and 0.01% NaN3. Fluorochrome-conjugated monoclonal antibodies were used for staining, along with a mouse Fc receptor blocking agent (clone: 93). Fixation and permeabilization were performed using an eBioscience/Thermo Fisher kit. Flow cytometric analysis was conducted to identify specific cell populations, including M2 macrophages (CD45+CD11b+F4/80+CD206+), myeloid-derived suppressor cells (MDSCs, CD45+CD11b+Gr1+), regulatory T cells (Tregs, CD45+CD4+FoxP3+CD25+), and CD8+ T cells (CD45+CD11b−CD8+). A BD FACS Canto II flow cytometer (Becton Dickinson, Mountain View, CA, USA) was utilized for this analysis, and data were processed using FlowJo V.10 software (FlowJo, LLC, Ashland, OR, USA). Antibodies used in the experiments included CD45 (clone 30-F11; 1:400 dilution), CD4 (clone GK1.5; 1:300 dilution), CD8a (clone 53-6.7; 1:400 dilution), CD25 (clone PC61; 1:300 dilution), CD11b (clone M1/70; 1:500 dilution), Gr1 (clone RB6-8C5; 1:400 dilution), CD69 (clone H1.2F3; 1:400 dilution), F4/80 (clone BM8; 1:500 dilution), and CD68 (clone FA-11; 1:400 dilution), all obtained from Biolegend (San Diego, CA, USA). For FoxP3 staining, a Foxp3 staining buffer set and FoxP3 antibody (clone 150D/E4) from eBioscience/Thermo Fisher (Grand Island, NY, USA) were used.

### 2.7. Statistical Analysis

All quantitative data were presented as mean ± SD and statistical significance (*p*) was calculated by a two-tailed Student’s t-test. For comparisons involving more than one group, a one-way ANOVA was performed, followed by Tukey’s multiple comparisons test.

## 3. Results

### 3.1. RAGE Pathway Activation in Pancreatic Cancer

**Figure 2:**
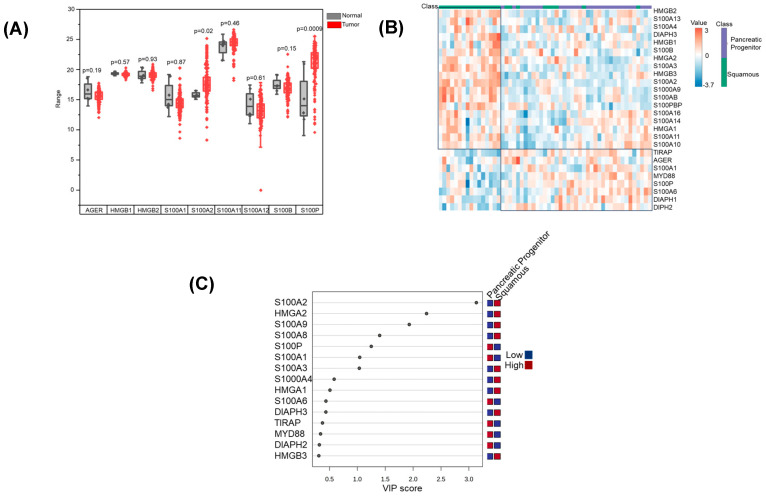
**Expression and clustering of RAGE (AGER) and its ligands in pancreatic cancer:** (**A**) Expression levels of RAGE (Receptor for Advanced Glycation End Products (encoded by the AGER gene)) and its ligands in pancreatic cancer patient samples obtained from The Cancer Genome Atlas (TCGA) database; (**B**) Clustering of RAGE ligands and activators within distinct pancreatic cancer subtypes (progenitor and squamous subtypes) as described by Bailey et al. [35]; (**C**) Variable Importance Plot (VIP) analysis highlights key ligands (e.g., S100A2, HMGA2) that differentiate the pancreatic progenitor and squamous subtypes. Red indicates upregulated genes, while blue indicates downregulated genes, underscoring subtype-specific mechanisms of RAGE pathway activation.

As an initial investigation, we sought to determine the relative expression of RAGE and its associated ligands in pancreatic cancer. Using data from pancreatic cancer patient samples from The Cancer Genome Atlas (TCGA), we demonstrated that although there was no change in the expression of RAGE, the RAGE ligands, S100P and S100A2, were significantly increased in pancreatic tumors when compared to normal pancreatic tissue (Figure 2A). We next determined if the RAGE pathway was implicated in a specific subtype of pancreatic cancer. An integrated genomic analysis of 456 pancreatic ductal adenocarcinomas identified four subtypes, with the squamous and pancreatic progenitor subtypes representing subtypes with the highest incidence and worst prognosis. These subtypes correlated with distinct histopathological characteristics and differential survival [39]. The squamous subtype was linked to squamous and adenosquamous histology, while the pancreatic progenitor subtype was associated with mucinous non-cystic adenocarcinomas and carcinomas arising from intraductal papillary mucinous neoplasms (IPMN). Notably, the squamous subtype was an independent poor prognostic factor [39]. Interestingly, using this database to evaluate the relative expression of RAGE-related proteins, clear clustering was observed, suggesting these two subtypes used distinct mechanisms for activating the RAGE pathway (Figure 2B). Variable importance plot (VIP) analysis identified S100A2, HMGA2, S100A9, and S100A8 as the primary ligands utilized by the pancreatic progenitor subtype, while S100P and S100A1 were the top ligands overexpressed in the squamous subtype (Figure 2C).

### 3.2. Azeliragon Inhibits RAGE Pathway Activation in Pancreatic Cancer Cell Lines

**Figure 3:**
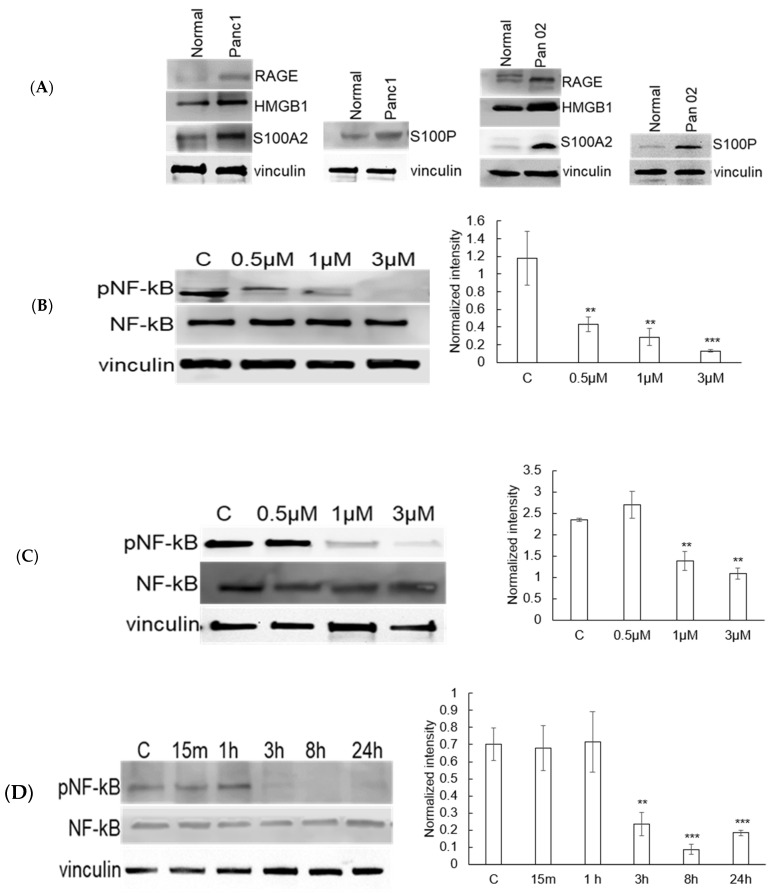

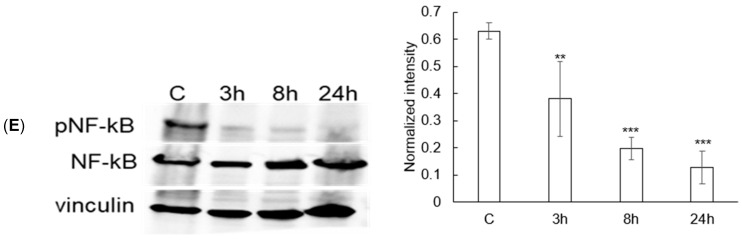
**Expression of RAGE and its ligands in pancreatic cancer cell lines and the inhibitory effect of Azeliragon on RAGE signaling:** (**A**) Expression levels of RAGE (Receptor for Advanced Glycation End Products) and its ligands—S100P, S100A2, and HMGB1—in human (Panc1) and murine (Pan02) pancreatic cancer cell lines; (**B**–**E**) Azeliragon (AZ), a RAGE inhibitor, inhibits the downstream signaling pathway of RAGE in pancreatic cancer cell lines: (**B**,**C**) Expression levels of phosphorylated NF-κB (pNF-κB) in Panc1 (**B**) and Pan02 (**C**) cells treated with indicated concentrations of AZ for 3 h; (**D**,**E**) Expression levels of pNF-κB in Panc1 (**D**) and Pan02 (**E**) cells treated with 1 µM AZ at indicated time points. Statistical significance: ** *p* < 0.01, *** *p* < 0.001 compared to the control group. The corresponding uncropped blots are provided in Supplementary Figures S2 and S3.

We next sought to determine if preclinical models of pancreatic cancer recapitulated the aberrant expression of RAGE pathway mediators observed in human tumors. Western blot analysis was done to evaluate the expression of RAGE and its associated ligands in both human (Panc1) and murine (Pan02) pancreatic cancer cell lines compared to normal pancreas. As shown in Figure 3A, in addition to RAGE expression, the expression of the RAGE ligands HMGB1, S100A2, and S100P was significantly upregulated in both human and murine pancreatic cancer cell lines when compared to normal pancreas. As we established the RAGE pathway is active in pancreatic cell lines grown in vitro, we next evaluated the potential for the RAGE inhibitor Azeliragon to modulate this pathway. Azeliragon demonstrated a dose-dependent inhibition of NF-κB phosphorylation, which represents a downstream mediator for RAGE pathway activation, with significant inhibition being observed at a drug concentration of 1 µM after 3 h in both Panc1 (Figure 3B) and Pan02 (Figure 3C) cell lines. We next performed a time course study in Panc1 cells, demonstrating pathway inhibition of Azeliragon (1 µM) as early as 3 h and was sustained for 24 h (Figure 3D). Pathway inhibition was also sustained for 24 h in the Pan02 line (Figure 3E), confirming the ability of Azeliragon to disrupt RAGE-mediated NF-κB activation in pancreatic cancer cell lines.

**Figure 4:**
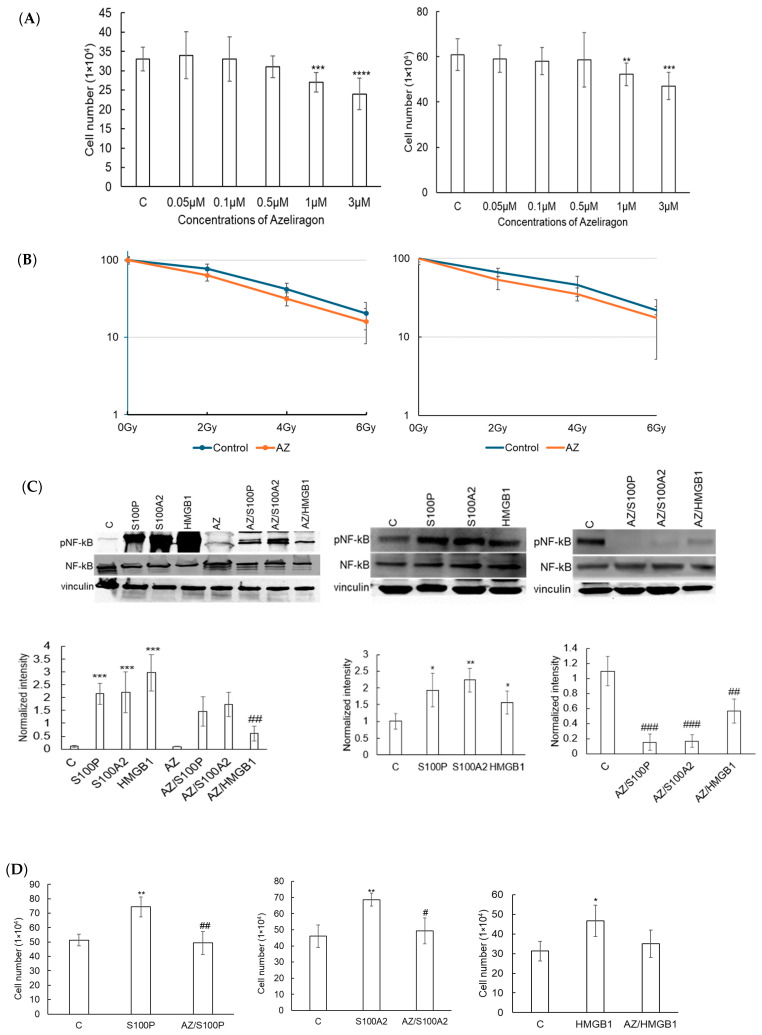

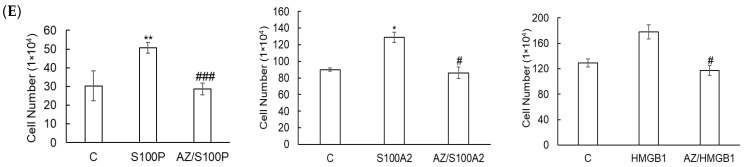
**Azeliragon inhibits proliferation and NF-κB activation in pancreatic cancer cell lines:** (**A**,**B**) Azeliragon (AZ) suppresses proliferation in pancreatic cancer cell lines: (**A**) Proliferation was determined by treating human Panc1 (left) and murine Pan02 (right) cells with indicated concentrations of AZ for 24 h. Live cells were counted using the trypan blue exclusion; (**B**) Clonogenic assay of Panc1 (left) and Pan02 (right) cells treated with 1 µM AZ for 24 h, followed by exposure to radiation doses of 2 Gy, 4 Gy, and 6 Gy. Results are representative of at least three independent experiments. Data represent mean ± standard deviation (SD). Significant results are indicated as: ** *p* = 0.01; *** *p* = 0.001; **** *p* < 0.001; (**C**) Azeliragon inhibits RAGE ligand-induced expression of phosphorylated NF-κB (pNF-κB). Expression level of pNF-κB in Panc1 (left) and Pan02 (right) cells stimulated with RAGE ligands (S100P, S100A2, HMGB1; 0.1 µM) for 24 h then treated with Azeliragon (1 µM AZ for 24 h). (**D**,**E**) Azeliragon inhibits RAGE ligand-induced cell proliferation. Effect of RAGE ligand stimulation (0.1 µM ligands for 24 h) on the proliferation of Panc1 (**D**) and Pan02 (**E**) cells with and without Azeliragon treatment (1 µM AZ for 24 h). Data represent mean ± SD of three or more independent experiments. Statistical significance: * *p* < 0.05, ** *p* < 0.01, *** *p* < 0.001 for ligands compared to the control group; ^#^ *p* = 0.05, ^##^ *p* < 0.01, ^###^ *p* = 0.001 for AZ-treated groups compared to their respective ligand-treated groups. The corresponding uncropped blots are provided in Supplementary Figure S4.

Next, we evaluated if Azeliragon-mediated inhibition of RAGE demonstrated anti-tumor activity in pancreatic cell lines in vitro. Despite potent inhibition of RAGE-mediated NF-κB activation, Azeliragon (1–3 µM) only led to modest inhibition of cell proliferation in both pancreatic cell lines tested (Figure 4A). In addition, no sensitization of radiation response was observed in these culture conditions (Figure 4B). We hypothesized that typical culture conditions when growing these cancer cell lines in vitro may not recapitulate the observed RAGE pathway activation in vivo. Specifically, RAGE ligands and their associated pathway activation may not be as pronounced when these lines are grown in standard culture conditions [40]. To test this, we stimulated cells with RAGE ligands observed to be upregulated in human pancreatic cancer, including S100P, S100A2, and HMGB1. Ligand stimulation led to potent NF-κB phosphorylation in Panc1 cells, which was completely attenuated when treated with Azeliragon (Figure 4C). When evaluating for proliferation, ligand stimulation led to an increase in proliferation, which, similar to western blot, was attenuated by Azeliragon (Figure 4D). Similar results were observed in the Pan02 line (Figure 4E). Collectively, we demonstrate the capacity of Azeliragon to inhibit the RAGE pathway in pancreatic cancer cell lines. However, determining the biological consequence of this pathway in vitro may be limited, which is likely attributed to the important role the tumor microenvironment plays in activating this pathway in vivo.

### 3.3. Azeliragon Demonstrates Anti-Tumor Activity and Modulates the Immune Microenvironment in Pancreatic Cancer Cell Lines In Vivo

**Figure 5:**
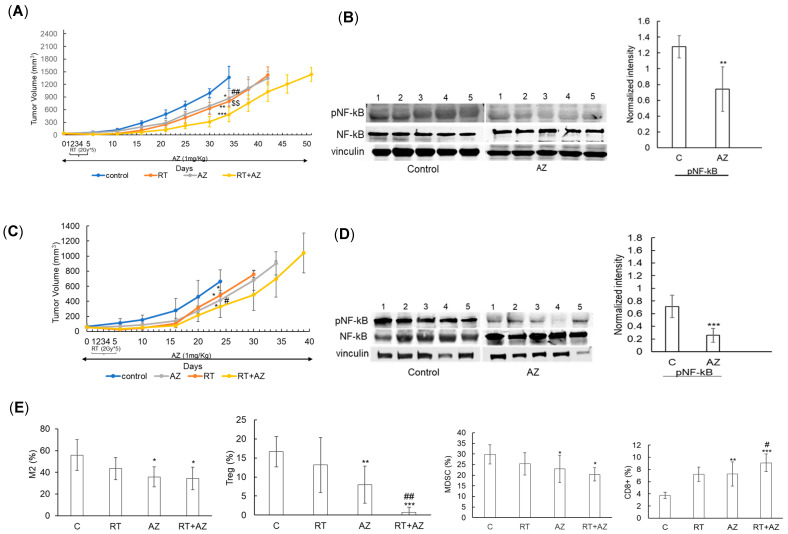
**The in vivo antitumor activity of Azeliragon in pancreatic cancer mouse models:** (**A**,**B**) Panc1 cells were injected into the flank of NU/NU mice. On day 14 post-tumor implantation, AZ treatment (1 mg/kg, i.p.) was initiated, and one day later, RT treatment (2 Gy X 5 days) was initiated. AZ treatment continued daily: (**A**) Tumor growth curves of mice. Data represent mean ± SD. Statistical significance calculated on day 34: * *p* < 0.05, ** *p* < 0.01, *** *p* < 0.001 compared to the control group; ^##^ *p* < 0.01 RT + AZ significantly different from the AZ group; ^$$^ *p* < 0.01 RT + AZ significantly different from the RT group. For correlative studies, mice were euthanized 10 days after the first day of treatment, and tumors were harvested for further analysis; (**B**) Expression levels of phosphorylated NF-κB (pNF-κB) in tumors in indicated treatment groups; (**C**–**E**): Pan02 cells were injected subcutaneously into the flank of C57BL/6 mice. On day 14 post-tumor implantation, AZ treatment (1 mg/kg) was initiated, and one day later, RT treatment (2 Gy X 5 days) was initiated. AZ treatment continued daily until the mice met the criteria requiring euthanization; (**C**) Tumor growth of C57BL/6 mice. Data represents mean ± SD. Statistical significance as calculated on day 24: * *p* < 0.01 significantly different from the control group. ^#^ *p* < 0.05 RT + AZ significantly different from RT group. For correlative studies, tumors were harvested 24 days post-tumor implantation; (**D**) Expression of pNF-kB in tumors in indicated treatment groups; (**E**) Immune profiling of tumors was performed using flow cytometry. Data represents mean ± SD. * *p* < 0.05, ** *p* < 0.01, *** *p* < 0.001 significantly different from control group. ^#^ *p* < 0.05, ^##^ *p* < 0.01 significantly different from RT group. The corresponding uncropped blots are provided in Supplementary Figure S4.

In vivo studies were conducted to assess the anti-tumor activity of Azeliragon, both alone and in combination with radiation therapy (RT), in pancreatic cancer mouse models. Panc1 cells were injected into the flank of NU/NU mice, and treatment with Azeliragon (1 mg/kg, i.p.) was initiated 14 days post-tumor implantation. Daily RT (2 Gy × 5 days) began one day after Azeliragon treatment. Azeliragon was well tolerated in mice and did not result in significant weight loss (Supplementary Figure S1). Mice treated with Azeliragon alone (*p* < 0.05) or RT alone (*p* < 0.01) exhibited tumor growth delay compared to controls (Figure 5A). The combination of Azeliragon and RT led to an additive tumor growth delay when compared to control or either treatment alone (*p* < 0.001 for RT+AZ vs. control, *p* < 0.01 for RT + AZ vs. AZ alone, *p* < 0.01 for RT + AZ vs. RT alone). Mice treated with Azeliragon and RT survived beyond day 50, while all control mice reached endpoint criteria by day 35. Correlative studies demonstrated target inhibition of Azeliragon in vivo, determined by decreased NF-κB phosphorylation (Figure 5B). Similar findings were observed when we extended these studies to the murine pancreatic cancer cell line Pan02 (Figure 5C,D). Using this model, we went on to determine the immune consequences of Azeliragon. Interestingly, Azeliragon-mediated RAGE inhibition modulated several tumor-supportive, immune-suppressive cells within the pancreatic cancer microenvironment (Figure 5E). This included a reduction in M2 macrophages and Tregs, which were further diminished when combined with RT. The combination of Azeliragon and RT led to a decrease in MDSCs. This reduction in immune suppression was coupled with an increase in immune activation in Azeliragon-treated mice, which was determined by an increase in CD8+ T cells. Collectively, these findings support the role Azeliragon plays in targeting both pancreatic cancer cells and this tumor’s immune suppressive microenvironment.

## 4. Discussion

Our study demonstrates that RAGE inhibition using Azeliragon leads to anti-tumor and immunomodulatory effects in preclinical models of pancreatic cancer, with an additive growth delay observed when combined with radiation therapy. Pancreatic cancer is highly resistant to standard therapies, largely due to overexpression of anti-apoptotic proteins and an immunosuppressive microenvironment, mechanisms that promote cancer cell survival and spread [3,4,5]. These characteristics are closely associated with NF-κB activation, a downstream target of RAGE signaling. In our study, Azeliragon effectively inhibited RAGE-mediated NF-κB activation, thus impacting key pathways associated with tumor progression and therapeutic resistance.

Consistent with prior literature, we observed significantly elevated levels of RAGE ligands—HMGB1, S100A2, and S100P—in both human and murine pancreatic cancer cell lines relative to normal pancreatic tissue [21]. Azeliragon effectively suppressed RAGE-mediated NF-κB activation in both in vitro and in vivo settings. Although the drug exhibited only a modest impact on cell proliferation and radiosensitivity in standard in vitro culture, stimulation with RAGE ligands under in vitro conditions led to increased proliferation, which was completely inhibited by Azeliragon. This suggests that the modest impact observed in traditional culture conditions may stem from the lack of a complex microenvironment typically present in vivo. Our in vivo studies underscored Azeliragon’s anti-tumor efficacy, both as monotherapy and in combination with RT. Mice treated with both Azeliragon and RT demonstrated delayed tumor growth and an extended survival beyond 50 days, compared to control mice, which succumbed by day 35. While other RAGE inhibitors, such as cromolyn and a S100P-derived inhibitor, have shown anti-tumor effects in pancreatic cancer models, their mechanisms are often limited to binding specifically to S100P, which restricts their utility against other RAGE ligands involved in pancreatic cancer progression [15,32,33]. In contrast, Azeliragon’s ability to bind directly to RAGE and inhibit multiple ligands of RAGE offers a broader inhibition of the RAGE pathway, which is advantageous given that multiple ligands are implicated in promoting tumor growth, migration, and resistance to therapy via the RAGE pathway [7,18,21,34].

A notable aspect of Azeliragon’s mechanism is its immune-modulatory capacity. Pancreatic cancer is characterized by a highly immunosuppressive environment, populated by Tregs, MDSCs, and M2 macrophages that support tumor growth and hinder immune-mediated tumor destruction [5]. In our study, Azeliragon treatment notably reduced these suppressive cell populations while increasing cytotoxic CD8+ T cell infiltration, thereby reshaping the immune environment towards a pro-inflammatory, anti-tumor state. Although our study did not directly explore the mechanism by which Azeliragon modulates the tumor immune microenvironment, this effect is likely mediated through the inhibition of RAGE-driven NF-κB signaling. NF-κB has been shown to influence the recruitment and differentiation of immune cells, including driving the polarization of macrophages toward the M2 phenotype [41,42,43,44]. Additionally, RT promotes immunogenic cell death and increases CD8+ T cell infiltration, and thus, the combined use of Azeliragon and RT synergistically creates a tumor microenvironment potentially more amenable to immune-mediated eradication [36,45,46].

The clinical implications of these findings suggest that Azeliragon, through its dual targeting of the RAGE pathway and immune-suppressive microenvironment, could be a promising candidate in the treatment of pancreatic cancer. Given that Azeliragon has shown safety in human trials for Alzheimer’s disease, its repurposing for pancreatic cancer could expedite clinical translation. Our findings support further clinical studies to confirm Azeliragon’s efficacy as a monotherapy and in combination with RT in patients with pancreatic cancer. Additionally, given the immunomodulatory effects observed, it may be valuable to explore Azeliragon in combination with immune checkpoint inhibitors to further enhance T cell-mediated anti-tumor activity.

While the current study focuses on the role of Azeliragon in pancreatic cancer, the involvement of RAGE in other cancers, such as colorectal and lung cancer, suggests that Azeliragon could have broader therapeutic applications [14,47]. Future studies should explore its effectiveness in these cancers to determine its potential clinical utility across malignancies and evaluate whether cross-cancer side effects might arise. Such investigations could further inform the safety profile and optimize the use of Azeliragon in oncology. Additionally, as the only RAGE inhibitor currently cleared for human use, Azeliragon presents a unique opportunity to benchmark its therapeutic advantages against other agents targeting RAGE, both in preclinical and clinical settings. Such comparisons would not only elucidate its distinctive benefits but also contribute to optimizing treatment regimens for pancreatic cancer.

Although the findings of this study provide evidence of Azeliragon’s therapeutic potential in pancreatic cancer, some limitations are acknowledged. First, while the use of murine models allows for the controlled study of Azeliragon’s effects on tumor growth and the tumor microenvironment, the generalizability of these results to human patients requires further validation in clinical settings. The complexities of human pancreatic cancer, including genetic heterogeneity and patient-specific tumor microenvironment dynamics, may influence treatment efficacy. Second, long-term studies are needed to evaluate its sustained efficacy and potential toxicity, including possible off-target effects and side effects that may arise with chronic use. While Azeliragon has demonstrated a favorable safety profile in clinical trials for Alzheimer’s disease, with patients receiving the drug for up to 18 months, its safety in the context of cancer therapy, particularly when combined with radiation or other treatments, warrants further investigation [48].

## 5. Conclusions

Our study demonstrates the therapeutic potential of Azeliragon, a RAGE inhibitor, as an adjunctive treatment in pancreatic cancer. Azeliragon effectively inhibited NF-κB signaling, a pathway critical to RAGE-mediated tumor progression and therapeutic resistance, resulting in significant tumor growth delay alone and when combined with radiation therapy in preclinical models. Additionally, Azeliragon displayed immunomodulatory effects within the tumor microenvironment, reducing immune-suppressive cells while enhancing CD8+ T cell infiltration. This dual action of direct tumor inhibition and immune modulation suggests that Azeliragon holds promise for improving pancreatic cancer outcomes. Clinical studies are warranted to validate these findings and explore Azeliragon’s integration into treatment regimens to improve pancreatic cancer outcomes.

## Data Availability

Data are available on request from the corresponding author.

## Supplementary Materials

The following supporting information can be downloaded at: https://www.mdpi.com/article/10.3390/cancers17010017/s1, Figure S1: Body weight monitoring of mice during study. Figure S2: Uncropped western blots showing expression of RAGE and its ligands in pancreatic cancer cell (Figure 3A in manuscript). Figure S3: Uncropped western blots showing the inhibitory effect of Azeliragon on RAGE signaling (Figure 3B-E in manuscript). Figure S4: Uncropped western blots showing antitumor activity of Azeliragon in vitro and in vivo (Figure 4C and Figure 5B&D in manuscript).
