# Supplementary material for: The RAGE Inhibitor TTP488 (Azeliragon) Demonstrates Anti-Tumor Activity and Enhances the Efficacy of Radiation Therapy in Pancreatic Cancer Cell Lines
Source: Cancers (Basel). 2024 Dec 24;17(1):17. doi: 10.3390/cancers17010017 (PMC11718873; doi:10.3390/cancers17010017)
Supplement: Supplementary file 1 [file cancers-17-00017-s001.zip › cancers-3370096-supplementary.pptx]

## Slide 1
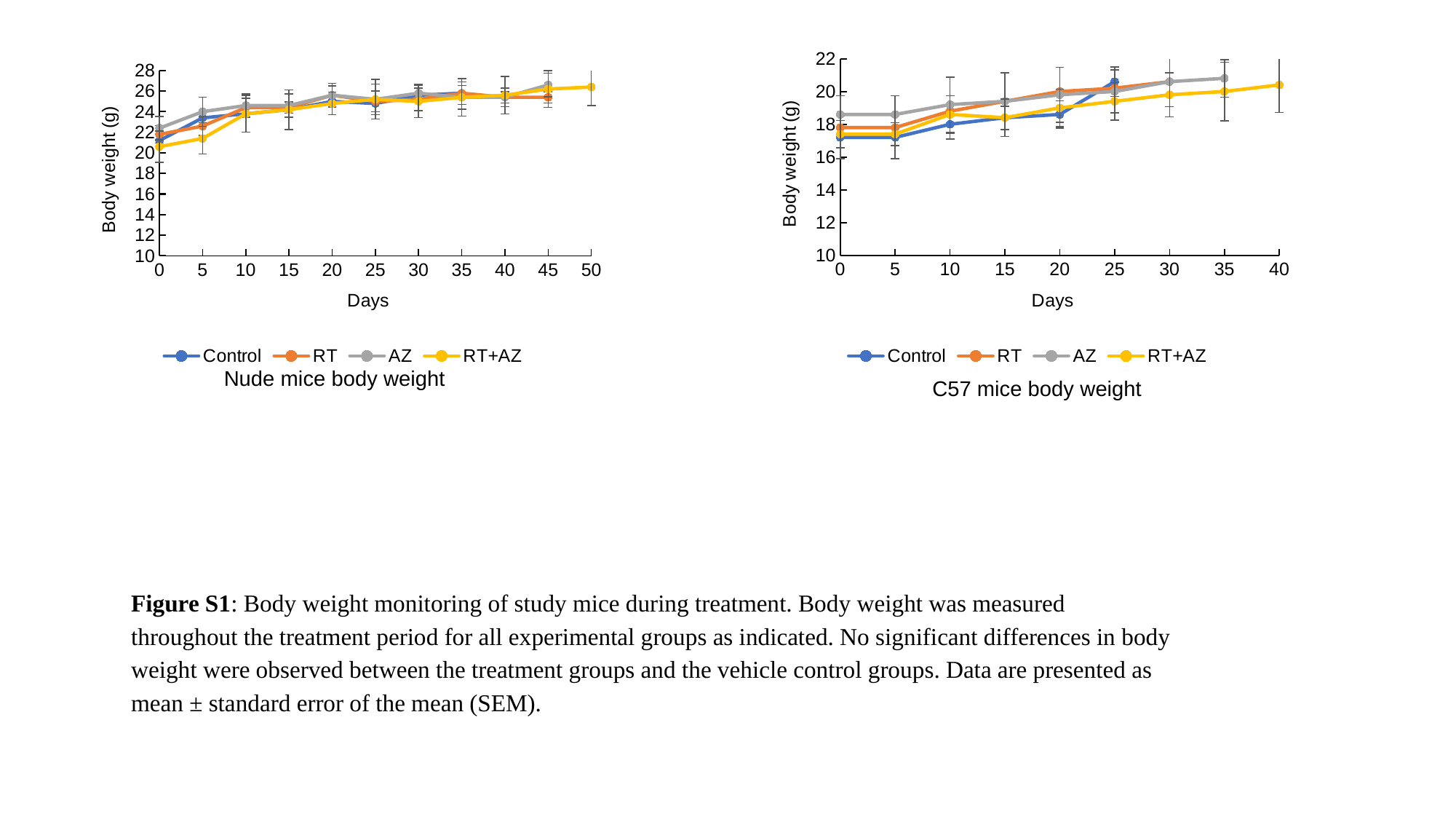

### Chart
| Category | Control | RT | AZ | RT+AZ |
|---|---|---|---|---|
| 0 | 17.2 | 17.8 | 18.6 | 17.4 |
| 5 | 17.2 | 17.8 | 18.6 | 17.4 |
| 10 | 18.0 | 18.8 | 19.2 | 18.6 |
| 15 | 18.4 | 19.4 | 19.4 | 18.4 |
| 20 | 18.6 | 20.0 | 19.8 | 19.0 |
| 25 | 20.6 | 20.2 | 20.0 | 19.4 |
| 30 | None | 20.6 | 20.6 | 19.8 |
| 35 | None | None | 20.8 | 20.0 |
| 40 | None | None | None | 20.4 |
### Chart
| Category | Control | RT | AZ | RT+AZ |
|---|---|---|---|---|
| 0 | 21.2 | 21.8 | 22.4 | 20.6 |
| 5 | 23.4 | 22.6 | 24.0 | 21.4 |
| 10 | 23.8 | 24.4 | 24.6 | 23.8 |
| 15 | 24.2 | 24.4 | 24.6 | 24.2 |
| 20 | 25.0 | 25.6 | 25.6 | 24.8 |
| 25 | 24.8 | 25.0 | 25.2 | 25.2 |
| 30 | 25.6 | 25.2 | 25.8 | 25.0 |
| 35 | 25.8 | 25.8 | 25.4 | 25.4 |
| 40 | None | 25.4 | 25.4 | 25.6 |
| 45 | None | 25.4 | 26.6 | 26.2 |
| 50 | None | None | None | 26.4 |Nude mice body weight
C57 mice body weight
Figure S1: Body weight monitoring of study mice during treatment. Body weight was measured throughout the treatment period for all experimental groups as indicated. No significant differences in body weight were observed between the treatment groups and the vehicle control groups. Data are presented as mean ± standard error of the mean (SEM).

## Slide 2
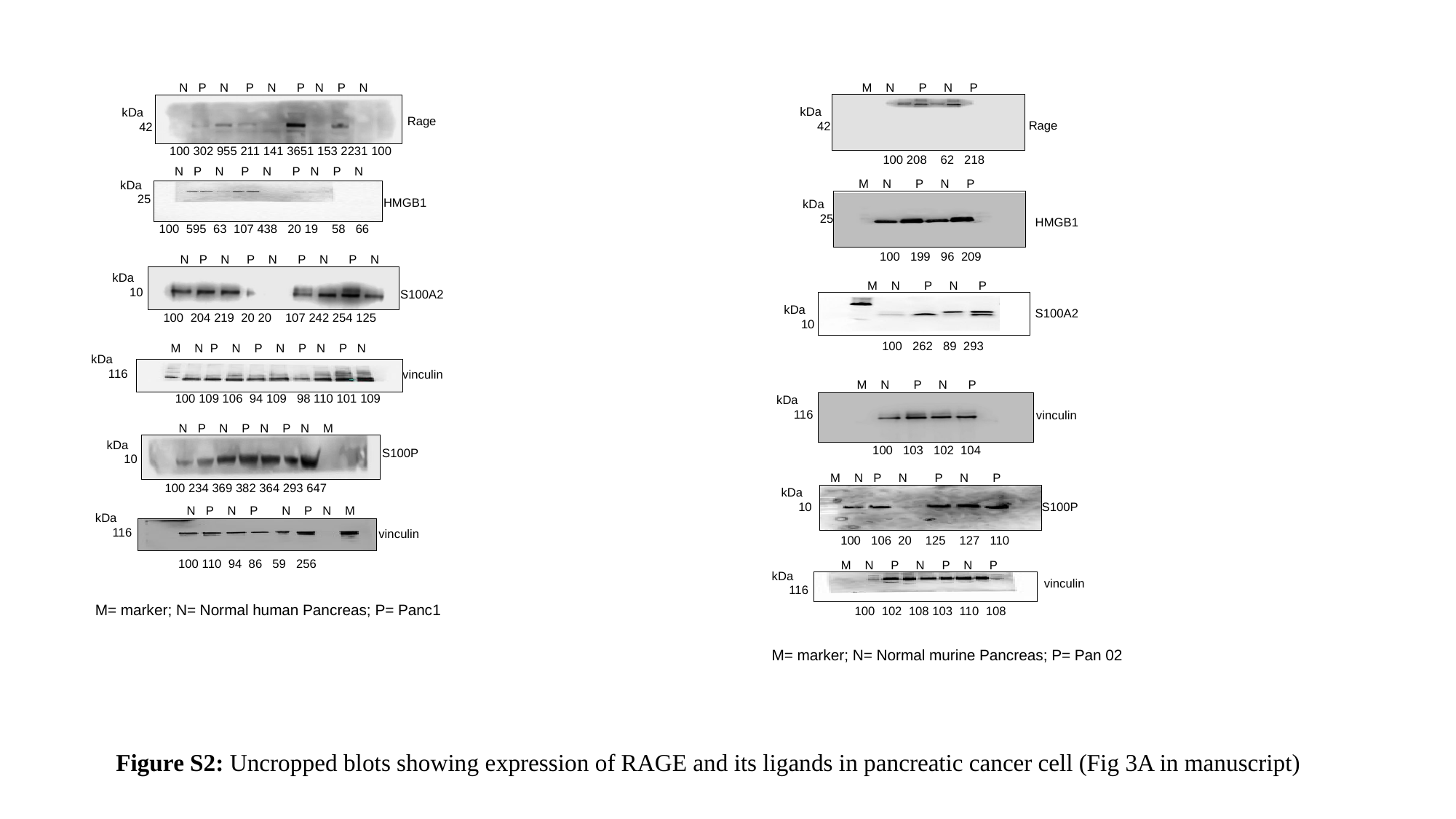

N P N P N P N P N
kDa
 42
Rage
100 302 955 211 141 3651 153 2231 100
M N P N P
kDa
 42
Rage
100 208 62 218
 N P N P N P N P N
kDa
 25
HMGB1
100 595 63 107 438 20 19 58 66
M N P N P
kDa
 25
HMGB1
100 199 96 209
 N P N P N P N P N
kDa
 10
S100A2
100 204 219 20 20 107 242 254 125
M N P N P
kDa
 10
S100A2
100 262 89 293
M N P N P N P N P N
kDa
 116
vinculin
100 109 106 94 109 98 110 101 109
M N P N P
kDa
 116
vinculin
100 103 102 104
 N P N P N P N M
kDa
 10
S100P
100 234 369 382 364 293 647
M N P N P N P
kDa
 10
S100P
100 106 20 125 127 110
 N P N P N P N M
kDa
 116
vinculin
100 110 94 86 59 256
M N P N P N P
kDa
 116
vinculin
100 102 108 103 110 108
M= marker; N= Normal human Pancreas; P= Panc1
M= marker; N= Normal murine Pancreas; P= Pan 02
Figure S2: Uncropped blots showing expression of RAGE and its ligands in pancreatic cancer cell (Fig 3A in manuscript)

## Slide 3
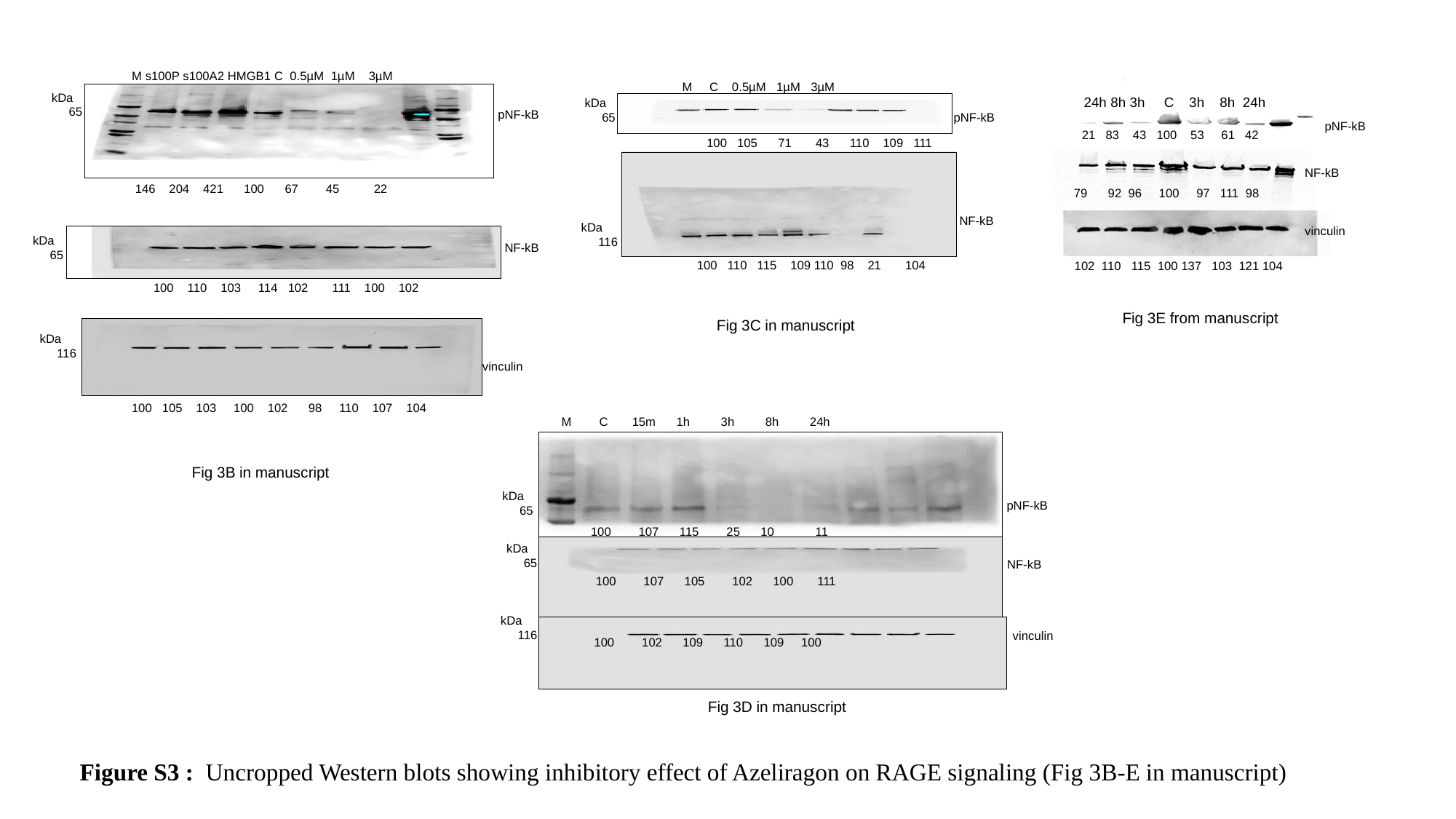

M s100P s100A2 HMGB1 C 0.5µM 1µM 3µM
kDa
 65
pNF-kB
 146 204 421 100 67 45 22
 M C 0.5µM 1µM 3µM
24h 8h 3h C 3h 8h 24h
pNF-kB
 21 83 43 100 53 61 42
NF-kB
 79 92 96 100 97 111 98
vinculin
 102 110 115 100 137 103 121 104
kDa
 65
pNF-kB
 100 105 71 43 110 109 111
NF-kB
kDa
 116
 100 110 115 109 110 98 21 104
kDa
 65
NF-kB
 100 110 103 114 102 111 100 102
 Fig 3E from manuscript
Fig 3C in manuscript
kDa
 116
vinculin
 100 105 103 100 102 98 110 107 104
 M C 15m 1h 3h 8h 24h
Fig 3B in manuscript
kDa
 65
pNF-kB
 100 107 115 25 10 11
kDa
 65
NF-kB
 100 107 105 102 100 111
kDa
 116
vinculin
 100 102 109 110 109 100
Fig 3D in manuscript
Figure S3 : Uncropped Western blots showing inhibitory effect of Azeliragon on RAGE signaling (Fig 3B-E in manuscript)

## Slide 4
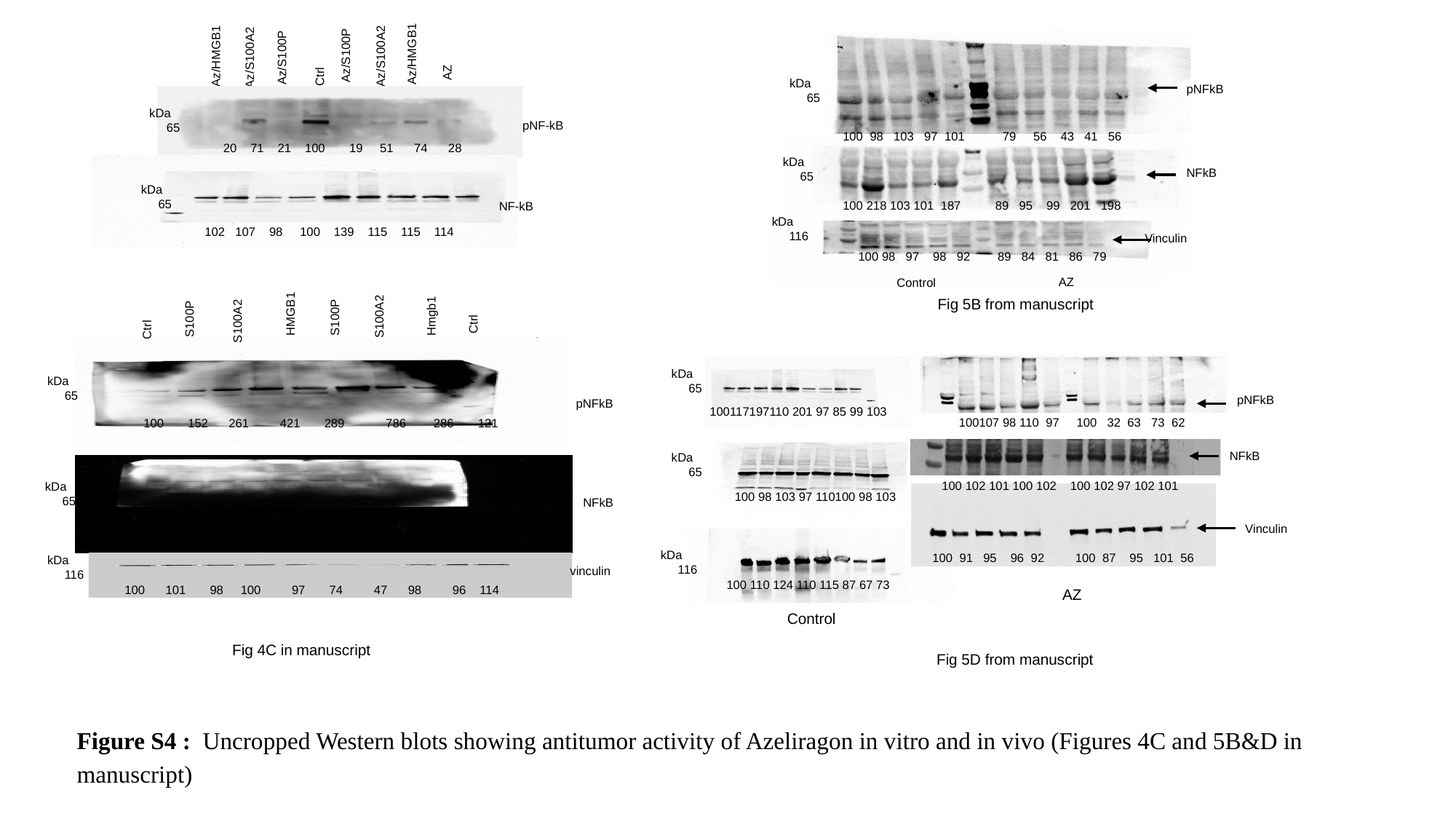

Az/S100P
 Az/HMGB1
 Az/HMGB1
 Az/S100A2
 Az/S100A2
 Ctrl
AZ
pNF-kB
 Az/S100P
pNFkB
NFkB
Vinculin
 AZ
 Control
 100 98 103 97 101 79 56 43 41 56
 100 218 103 101 187 89 95 99 201 198
 100 98 97 98 92 89 84 81 86 79
kDa
 65
 20 71 21 100 19 51 74 28
kDa
 65
NF-kB
 102 107 98 100 139 115 115 114
kDa
 65
kDa
 65
kDa
 65
kDa
 116
kDa
 116
 S100A2
HMGB1
 S100P
 Hmgb1
 S100P
 S100A2
 Ctrl
 Ctrl
pNFkB
NFkB
vinculin
 Fig 5B from manuscript
pNFkB
 100107 98 110 97 100 32 63 73 62
NFkB
 100 102 101 100 102 100 102 97 102 101
Vinculin
 100 91 95 96 92 100 87 95 101 56
 AZ
kDa
 65
kDa
 65
 100117197110 201 97 85 99 103
 100 152 261 421 289 786 286 121
kDa
 65
kDa
 65
 100 98 103 97 110100 98 103
kDa
 116
kDa
 116
 100 110 124 110 115 87 67 73
 100 101 98 100 97 74 47 98 96 114
 Control
Fig 4C in manuscript
 Fig 5D from manuscript
Figure S4 : Uncropped Western blots showing antitumor activity of Azeliragon in vitro and in vivo (Figures 4C and 5B&D in manuscript)
